# Potentially Inappropriate Prescriptions of Antipsychotics for Patients With Dementia

**DOI:** 10.3389/fphar.2021.695315

**Published:** 2021-05-31

**Authors:** Manuel Enrique Machado-Duque, Luis Fernando Valladales-Restrepo, Juan Alberto Ospina-Cano, María José Londoño-Serna, Jorge Enrique Machado-Alba

**Affiliations:** ^1^ Grupo de Investigación en Farmacoepidemiología y Farmacovigilancia, Universidad Tecnológica de Pereira-Audifarma S.A, Pereira, Colombia; ^2^ Grupo de Investigación Biomedicina, Facultad de Medicina, Fundación Universitaria Autónoma de Las Américas, Pereira, Colombia; ^3^ Semillero de Investigación en Farmacoepidemiología y Farmacovigilancia, Universidad Tecnológica de Pereira, Pereira, Colombia

**Keywords:** (MeSH): dementia, alzheimer disease, dementia vascular, antipsychotic agents, pharmacoepidemiology

## Abstract

Dementias are neurodegenerative and progressive diseases of the central nervous system. The objective of this study was to determine the frequency of potentially inappropriate prescriptions of antipsychotics in a group of patients diagnosed with dementia in Colombia. This was a cross-sectional study based on a population database for drug dispensing that identified prescriptions of antidementia drugs, antipsychotics, and other drugs for patients with a diagnosis of dementia. Descriptive statistics and bivariate and multivariate analyses were performed. A total of 11,372 patients with dementia were identified; 66.6% were women, and the mean age was 80.5 ± 9.6 years. Alzheimer’s disease was the most frequent diagnosis (76.6%). A total of 69.0% of patients received antidementia drugs. A total of 37.1% of patients received some antipsychotic, especially atypical antipsychotics (31.0%). Increased age, being treated with memantine, simultaneously presenting with anxiety, depression, and psychotic disorders, and concomitantly receiving anticonvulsants, bronchodilators and benzodiazepines were associated with a greater probability of being prescribed antipsychotics. More than one-third of patients with dementia received antipsychotic prescriptions, which are considered potentially inappropriate because they can worsen cognitive decline and favor the occurrence of adverse events.

## Introduction

Dementia is a neurodegenerative and progressive disease of the central nervous system characterized by chronic, global and generally irreversible deterioration of cognitive ability (Prince et al., 2013). Dementia may be due to a variety of underlying pathophysiological processes; the most common is Alzheimer’s disease (50–75%), followed by vascular dementia (20%), dementia with Lewy bodies (5%), and frontotemporal dementia (5%) (Cunningham et al., 2015).

Global rates of dementia are increasing rapidly, in line with population aging. The prevalence of dementia is 2–3% in the 70–75 age group, increasing to 25% in those over 85 (Rizzi et al., 2014). At advanced ages, women are more likely to develop dementia due to Alzheimer’s disease, while the prevalence of vascular dementia is higher in men (Rizzi et al., 2014).

The pathophysiological processes underlying dementia are not yet fully understood; however, it is known that all involve pathological protein accumulation (Kocahan and Doğan, 2017). These pathological accumulations are associated with synaptic failure, neuronal loss and atrophy that can have different anatomical distribution patterns (Frisoni et al., 2015).

Neuropsychiatric symptoms, including agitation, aggressiveness, paranoid delusions, and hallucinations, are widely prevalent among dementias, especially in the moderate and severe disease stages, which require either pharmacological or nonpharmacological intervention (Cerejeira et al., 2012). Very often, nonpharmacological management is sufficient to control symptoms, but sometimes, the severity of the disorder, the involvement in the patient, or the risk to oneself or others makes using drugs, including antipsychotics, necessary to control symptoms (Calsolaro et al., 2021). However, the Beers criteria and the STOPP/START criteria consider antipsychotics as potentially inappropriate prescriptions in this group of patients because they are associated with a greater number of adverse outcomes, such as cerebrovascular events and greater functional decline and mortality, and thus recommend avoiding their use unless there are no alternatives for the management of behavioral disorders or delirium (“American Geriatrics Society 2019 Updated AGS Beers Criteria(R) for Potentially Inappropriate Medication Use in Older Adults,” 2019; O'Mahony et al., 2015).

It has been shown that patients treated with antipsychotics can frequently present with anticholinergic side effects, such as orthostatic hypotension, confusion, drowsiness, cognitive impairment, and an increased risk of severe extrapyramidal effects that can be fatal (Schneider et al., 2006a; Schneider et al., 2006b; Calsolaro et al., 2021; Vina Latin Small Letter et al., 2021). In addition, the DART-AD trial found an increased risk of mortality in patients older than 65 years with dementia treated with any class of antipsychotics, both in the short term (12 weeks) and long term (over 48 months) (Hereu and Vallano, 2011).

The inadequate use of antipsychotics and the lack of precautions in their prescription for Colombian patients with different types of dementia are unknown. Therefore, the objective of this study was to determine the frequency of potentially inappropriate prescriptions of antipsychotics in a group of patients diagnosed with dementia in Colombia between October and December of 2019, as well as to identify the medications used, comorbidities and possible variables associated with the use of these antipsychotics in this older adult population.

## Materials and Methods

### Study Design and Patients

A cross-sectional study was conducted on the prescription patterns of antipsychotic drugs for patients diagnosed with dementia in out-patient setting. The data were obtained from a population database for drug dispensing that contains information on approximately 8.5 million people affiliated with the Health System of Colombia through six health insurance companies, corresponding to approximately 30.0% of the active affiliated population covered by the contributory or paid regime and 6.0% of the population covered by the state-subsidized regime, which serves 17.3% of the Colombian population.

Patients aged 50 years or older of either sex and seen as outpatients were selected in the main cities of Colombia, with a diagnosis of dementia who received a drug prescription in any month in the period from October 1 to December 31, 2019, were included (Medication for the treatment of any comorbidity or dementia in which an International Classification of Diseases (ICD-10) diagnostic code for dementia is used or registered). Identification was performed based on ICD-10 codes, considering the following diagnoses: Alzheimer’s disease (F000-F002, F009, G300, G301, G308 and G309), vascular dementia (F010-F013, F018 and F019), dementia in Parkinson’s disease (F023), dementia in Huntington’s disease (F022), dementia in Pick’s disease or frontotemporal dementia (F020), dementia in HIV (F024), dementia in Creutzfeldt-Jakob disease (F021) and unspecified dementias (F03X and F028).

The information of each dispensing and variables associated with the prescription is obtained from the pharmacies and the clinical registry, they are kept in a database with all the information and subsequently consulted through business intelligence, validated by validated by two members of the research group, identifying strange and missing values, until having a dataset with the included patients and being able to analyze it, and subsequently analyzed. Their drug dispensing records during the observation period were analyzed to identify the use of antipsychotics and all comedications. Those with incomplete information and those with two or more different dementia diagnoses were excluded.

### Variables

Based on the information on the consumption of drugs of the affiliated population, systematically obtained by the dispensing company (Audifarma SA), a database was designed that allowed collecting the following groups of variables: 1). Sociodemographic: sex, age, city, and geographic region of residence; 2). Clinical: type of dementia diagnosed and comorbidities. 3). Comorbidities: identified from the diagnoses reported using ICD-10 codes for the selected patients. They were categorized by number of comorbidities. The main cardiovascular, endocrine, rheumatologic, urological, renal, psychiatric, neurological, digestive, respiratory, and cancer diseases were identified; 4). Drugs for dementia treatment: acetylcholinesterase inhibitors (rivastigmine, donepezil, galantamine) and NMDA receptor antagonists (memantine); 5). Prescribed antipsychotics: Typical: chlorpromazine, fluphenazine, haloperidol, levomepromazine, pipotiazine, propericiazine, thioridazine, and trifluoperazine; and Atypical: amisulpride, aripiprazole, asenapine, clozapine, olanzapine, paliperidone, quetiapine, risperidone, sulpiride, and ziprasidone; and 6). Comedications were grouped into the following categories: 1) antidiabetics (oral and subcutaneous), 2) antihypertensives and diuretics, 3) lipid-lowering drugs; 4) antiulcers, 5) antidepressants, 6) benzodiazepines, 7) thyroid hormone, 8) anticonvulsants, 9) antiarrhythmics, 10) antihistamines, 11) bronchodilators and inhaled corticosteroids, 12) opioid analgesics, 13) nonopioid analgesics, 14) platelet antiaggregants, and 15) anticoagulants.

### Ethical Statement

The protocol was approved by the Bioethics Committee of Universidad Tecnológica de Pereira in the “research without risk” category (Code: 02–130420). The ethical principles established by the Declaration of Helsinki were respected. No personal data were collected from the patients.

### Data Analysis

The data were analyzed with the statistical package SPSS Statistics, version 26.0 for Windows (IBM, United States). A descriptive analysis was performed; qualitative variables are presented as frequencies and proportions, and quantitative variables are presented as measures of central tendency and dispersion. Quantitative variables were compared using Student’s t-test or ANOVA, and categorical variables were compared using the χ^2^ test. Binary logistic regression models were fitted using “prescribed antipsychotics” as the dependent variable; the covariates were the variables that were significantly associated with the dependent variable in the bivariate analyses and variables with biological plausibility to explain the outcome (prescription of an antipsychotic). A statistical significance level of *p* < 0.05 was adopted.

## Results

A total of 11,372 patients with a diagnosis of dementia were identified, distributed in 154 different cities or municipalities. The mean age was 80.5 ± 9.6 years (range: 50.0–105.1 years), and 66.6% (*n* = 7,573) were women. A total of 78.8% (*n* = 8,958) of the patients resided in capital cities, and the majority were in the Pacific Region (*n* = 3,009; 26.5%), followed by the Bogotá-Cundinamarca Region (*n* = 2,982; 26.2%), Caribbean Region (*n* = 2,758; 24.3%), Central Region (*n* = 2081; 18.3%), Eastern Region (*n* = 519; 4.6%) and Amazonia-Orinoquía Region (*n* = 23; 0.2%).

Most patients had a diagnosis of Alzheimer’s disease (*n* = 8,711; 76.6%), followed by unspecified dementia (*n* = 1767; 15.5%), vascular dementia (*n* = 657; 5.8%), dementia in Parkinson’s disease (*n* = 201; 1.8%), frontotemporal dementia (*n* = 21; 0.2%), dementia in Huntington’s disease (*n* = 9; 0.1%) and dementia in HIV (*n* = 6; 0.1%). A total of 69.0% (*n* = 7,841) were receiving pharmacological treatment for dementia, mainly rivastigmine (*n* = 4,843; 42.6%), followed by memantine (n = 3,756; 33.0%), donepezil (*n* = 164; 1.4%) and galantamine (*n* = 164; 1.4%).

Of the total number of patients identified, 4,222 (37.1%) had been prescribed at least one antipsychotic, distributed among 10 different antipsychotic drugs, with the use of atypical antipsychotics (*n* = 3,526, 31.0%) predominating over the use of typical antipsychotics (*n* = 1,023, 9.0%), with some patients receiving more than one of these drugs. Of the patients who were prescribed antipsychotics, 86.6% (*n* = 3,656) received a single drug, and 13.4% (*n* = 566) received two or more. The most commonly used antipsychotic was quetiapine, followed by levomepromazine and risperidone. The most commonly used pharmaceutical forms were tablets (*n* = 3,482; 30.6%), followed by oral solution (*n* = 1,026; 9.0%) and injectable solution (*n* = 82; 0.7%) (Table 1).

**TABLE 1:** Antipsychotics prescriptions for a group of patients with dementia in Colombia, 2019.

Antipsychotic	Frequency *n* = 11,372	%
Atypical	3,526	31.0
Quetiapine (tablet)	2,686	23.6
Risperidone	525	4.6
Tablet	399	3.5
Oral solution	120	1.1
Injectable	12	0.1
Clozapine (tablet)	312	2.7
Olanzapina (tablet)	185	1.6
Aripiprazol (tablet)	17	0.1
Paliperidone	10	0.1
Injectable	9	0.1
Tablet	1	0.0
Amisulpiride (tablet)	1	0.0
Typical	1,023	9.0
Levomepromazine	600	5.3
Oral solution	541	4.8
Tablet	63	0.6
Haloperidol	489	4.3
Oral solution	438	3.9
Injectable	57	0.5
Tablet	27	0.2
Pipothiazine (injectable)	6	0.1

### Comorbidities and Comedications

A total of 77.8% (*n* = 8,853) of all patients had some chronic disease. Of these, 42.9% (*n* = 3,805) had one disease, 31.8% (*n* = 2,812) had two diseases and 25.3% (*n* = 2,236) had three or more diseases. The 10 comorbidities that were most common in all patients were arterial hypertension (*n* = 4,922; 43.3%), diabetes mellitus (*n* = 1,547; 13.6%), urinary incontinence (*n* = 1,530; 13.5%), bipolar affective disorder (*n* = 993; 8.7%), anxiety disorders (*n* = 751; 6.6%), hypothyroidism (*n* = 769; 6.8%), depressive disorders (*n* = 751; 6, 6%), chronic kidney disease (*n* = 643; 5.7%), chronic obstructive pulmonary disease (*n* = 465; 4.1%) and benign prostatic hyperplasia (*n* = 434; 3.8%). Upon grouping, cardiovascular diseases were the most frequent (*n* = 5,169; 45.5%), followed by other psychiatric disorders (*n* = 2,644; 23.3%) and endocrine (*n* = 2,470; 21.7%), urological (*n* = 2029; 17.8%), neurological (*n* = 1,059; 9.3%), rheumatic (*n* = 776, 6.8%), gastrointestinal (*n* = 602; 5.3%) and respiratory (*n* = 501; 4.4%) diseases.

The most prescribed comedications were antihypertensives and diuretics (*n* = 6,902, 60.7%), followed by antidepressants (*n* = 5,198; 45.7%), nonopioid analgesics (*n* = 3,696; 32.5%), antiulcers (*n* = 3,384; 29.8%), platelet antiaggregants (*n* = 3,125; 27.5%), lipid-lowering drugs (n = 2,915; 25.6%), thyroid hormone (*n* = 2,558; 22.5%), oral and subcutaneous antidiabetic drugs and insulins (*n* = 2004; 17.6%), anticonvulsants (*n* = 1,491; 13.1%) and bronchodilators and inhaled corticosteroids (*n* = 1,416; 12.5%).

### Bivariate Comparisons by Type of Dementia

Alzheimer’s disease, unspecified dementia and vascular dementia predominated in women and occurred at a higher mean age. Patients with vascular dementia and Parkinson's disease presented with comorbidities with greater frequency. Rivastigmine and memantine were the most commonly used antidementia drugs for all types of dementia, especially Alzheimer’s disease. The prescription of antipsychotics was more common for patients with vascular dementia, with the use of atypical antipsychotics prevailing for all types of dementia; the lowest frequencies of use of typical antipsychotics were found for patients with dementia in Parkinson’s disease. The use of antihypertensives and diuretics predominated for all patients, while platelet antiaggregants were prescribed more for patients with vascular dementia (Table 2).

**TABLE 2:** Comparison of sociodemographic, clinical and pharmacological variables with types of dementia in a group of patients in Colombia, 2019.

Variables	Alzheimer’s disease		Unspecified dementias		Vascular dementia		Dementia in Parkinson’s disease		Other dementias	
	*n* = 8,711	%	*n* = 1767	%	*n* = 657	%	*n* = 201	%	*n* = 36	%
Woman	5,945	68.2	1,151	65.1	375	57.1	87	43.3	15	41.7
Age (mean; SD)	80.7 ± 9.5		80.5 ± 10.1		80.9 ± 9.7		77.7 ± 9.5		68.4 ± 9.6	
Geographic regions	–	–	–	–	–	–	–	–	–	–
Pacific region	2045	23.2	599	33.9	299	45.5	75	37.3	11	30.6
Bogotá-cundinamarca region	2,304	26.4	523	29.6	110	16.7	34	16.9	11	30.6
The caribbean region	2,332	26.8	297	16.8	83	12.6	36	17.9	10	27.8
Central region	1,591	18.3	298	16.9	145	22.1	46	22.9	1	2.8
Eastern region	444	5.1	46	2.6	17	2.6	9	4.5	3	8.3
Amazon region-orinoquía	15	0.2	4	0.2	3	0.5	1	0.5	0	0.0
With chronic comorbidities	6,748	77.5	1,349	76.3	559	85.1	170	84.6	27	75.0
1 pathology	2,914	33.5	595	33.7	232	35.3	52	24.9	12	33.3
2 pathologies	2,132	24.5	426	24.1	175	23.1	70	34.8	9	25.0
≥3 pathologies	1702	19.5	328	18.6	152	23.1	48	23.9	6	16.7
Cardiovascular pathologies	3,998	45.9	776	43.9	314	47.8	71	35.3	10	27.8
Psychiatric pathologies	2013	23.1	407	23.0	155	23.6	56	27.9	13	36.1
Endocrine pathologies	1924	22.1	365	20.7	147	22.4	30	14.9	4	11.1
Urological pathologies	1,533	17.6	285	16.1	159	24.2	47	23.4	5	13.9
Neurological pathologies	696	8.0	170	9.6	95	14.5	91	45.3	7	19.4
Use of antidementiants	6,571	75.4	897	50.8	314	47.8	50	24.9	9	25.0
Rivastigmine	3,917	45.0	686	38.8	192	29.2	42	20.9	6	16.7
Memantine	3,372	38.7	238	13.5	134	20.4	8	4.0	4	11.1
Donepezil	146	1.7	13	0.7	5	0.8	0	0.0	0	0.0
Galantamine	134	1.5	21	1.2	8	1.2	1	0.5	0	0.0
Use of antipsychotics	3,139	36.0	712	40.3	279	42.5	70	34.8	22	61.1
Atypical	2,680	30.8	537	30.4	226	34.4	66	32.8	17	47.2
Quetiapine	2031	23.3	416	23.5	178	27.1	49	24.4	12	33.3
Risperidone	447	5.1	52	2.9	18.0	2.7	6	3.0	2	5.6
Clozapine	217	2.5	61	3.5	21	3.2	10	5.0	3	8.3
Typical	705	8.1	232	13.1	74	11.3	5	2.5	7	19.4
Levomepromazine	427	4.9	127	7.2	37	5.6	5	2.5	4	11.1
Haloperidol	314	3.6	129	7.3	42	6.4	1	0.5	3	8.3
Pipothiazine	4	0.0	2	0.1	0	0.0	0	0.0	0	0.0
Comedications	–	–	–	–	–	–	–	–	–	–
Antihypertensives and diuretics	5,267	60.5	1,078	61.0	439	66.8	103	51.2	15	41.7
Antidepressants	3,922	45.0	851	48.2	321	48.9	88	43.8	16	44.4
Non-opioid pain relievers	2,786	32.0	593	33.6	228	34.7	77	38.3	12	33.3
Antiulcer	2,517	28.9	572	32.4	214	32.6	67	33.3	14	38.9
Platelet antiaggregants	2,353	27.0	472	26.7	250	38.1	44	21.9	6	16.7

*Other dementias: Includes dementia in Huntington’s disease, frontotemporal dementia, and dementia in human immunodeficiency virus infection.

### Multivariate Analysis

Binary logistic regression analysis was performed to determine the factors associated with the use of antipsychotics for patients diagnosed with dementia. Female sex, diagnosis of Alzheimer-type dementia, and being treated with rivastigmine decreased the probability of being prescribed an antipsychotic. Increased age, being treated with memantine, presenting concomitantly with anxiety, depression, and psychotic disorders, and receiving anticonvulsants, bronchodilators and benzodiazepines simultaneously were associated with a greater probability of being prescribed antipsychotics (Table 3).

**TABLE 3:** Multivariate analysis of the variables associated with the prescription of antipsychotics for a group of patients with dementia in Colombia, 2019.

Variable	*p*	ORa	95% CI	
			Lower	Upper
Female gender	<0.01	0.814	0.745	0.889
Age less than 65 years	Ref	Ref		
Age between 65 and 74 years	<0.01	1.398	1.153	1.694
Age between 75 and 84 years	<0.01	1.656	1.388	1.975
Age greater than or equal to 85 years	<0.01	1.916	1.604	2.29
Be treated in Bogotá D.C/Cundinamarca	0.004	0.856	0.771	0.95
Diagnosis of Alzheimer’s dementia	0.009	0.86	0.769	0.963
Being treated for dementia with rivastigmine	<0.01	0.835	0.764	0.913
Being treated for dementia with memantine	0.017	1.121	1.021	1.232
Anxiety (comorbidity)	<0.01	13.107	10.783	15.93
Depression (comorbidity)	<0.01	2.184	1.853	2.574
Osteoporosis (comorbidity)	0.014	0.661	0.475	0.919
Psychotic disorders (comorbidity)	<0.01	10.273	6.961	15.162
Concomitant use of antiepileptic drugs	<0.01	1.9	1.686	2.142
Concomitant use of benzodiazepines	<0.01	1.946	1.644	2.302
Concomitant use of bronchodilators	0.001	1.237	1.092	1.403
Concomitant use of lipid-lowering drugs (statins)	<0.01	0.839	0.761	0.924

Binary logistic regression: adjusted for sex, age, place of treatment, type of dementia, treatment of dementia, comorbidities, and comedications. ORa, Adjusted Odds Ratio; CI95%, Confidence interval.

## Discussion

The present study was able to determine the prescription and the potentially inappropriate use of antipsychotics for patients diagnosed with dementia in six health insurance entities in Colombia. The results of this study are of great interest because a high frequency of use of antipsychotics was found in this specific group of patients, who may be at risk of adverse events. With these results, a baseline for the problem is established, which may be useful for making better informed decisions and improving the use of these drugs.

The mean age of the analyzed patients was close to 80 years, consistent with what has been described in different studies conducted worldwide, i.e., a typical age of presentation of dementias above 65 years (Prince et al., 2013; Arvanitakis et al., 2019). Regarding the sex of the patients, approximately two-thirds of the study population were women, which is also consistent with what has been described in the world population (Goodman et al., 2017; Valladales-Restrepo et al., 2019). The most prevalent type of dementia was Alzheimer’s disease, a finding that agrees with reports on the prevalence of dementia worldwide and imparts confidence and validity to the results of this study (Cunningham et al., 2015). Additionally, the most commonly used antidementia drug in the evaluated patients was rivastigmine, agreeing with the data reported in different studies (Jia et al., 2016; Calvo-Torres et al., 2019). In addition, a diagnosis of Alzheimer-type dementia acted as a protective factor for the prescription of antipsychotics, unlike that seen for other types of dementia, a finding that may be explained by the difference in the frequency of psychotic symptoms, agitation and insomnia among the different causes of dementia (Collins et al., 2020).

It was found that more than one-third of patients diagnosed with dementia were prescribed at least one antipsychotic, putting these patients at particular risk of adverse reactions and worsening of symptoms, a result that is similar to those reported in studies conducted in Norway by Langballe et al. (2013) and in Germany by Schulze et al. (2012) (Schulze et al., 2013; Langballe et al., 2014), who found a frequencies of use of 30.4 and 39.8%, respectively. Additionally, it is noteworthy that there was a predominance of prescriptions for atypical antipsychotics over typical antipsychotics, a relationship that contrasts with results from studies by Schulze et al. and Langballe et al., in which 24.4 and 19.5% of patients with dementia received typical antipsychotics, respectively (Schulze et al., 2013; Langballe et al., 2014).

The most commonly used antipsychotic was quetiapine; in contrast, in the United States, the use of olanzapine predominates, and in Norway, the use of risperidone predominates (Kamble et al., 2009; Langballe et al., 2014; Maust et al., 2015). This trend in Colombia may be due to the off-label use of quetiapine as a sedative-hypnotic (Dolder and McKinsey, 2010).

Cardiovascular disease was the most prevalent comorbidity in almost half of patients with Alzheimer’s disease. It has been described that the presence of systolic hypertension can be a modifiable risk factor for the onset of cognitive decline and vascular dementia and that its control with antihypertensive treatment reduces the development of Alzheimer’s disease, as well as of other dementias, in the future (Gorelick et al., 2011). In addition, it is noteworthy that coronary and atherosclerotic carotid disease are risk factors for the onset of cognitive decline and vascular dementia (Rosano et al., 2005; Zhong et al., 2012).

The association of having a diagnosis of osteoporosis with a lower probability of being prescribed an antipsychotic in this group of patients with dementia contrasts with the results of a study by Crews et al. (2012), which found a significant relationship between the use of antipsychotics and a decrease in bone mineral density (Crews and Howes, 2012) as well as a slightly higher risk of having a hip fracture (Jalbert et al., 2010), which should serve as an alert to avoid their use in these patients. In turn, the use of lipid-lowering drugs was also associated with a lower risk of receiving antipsychotics, which supports the findings by Shen et al., who showed that statins can help in the treatment of schizophrenia, based on reduced scores on the positive symptoms, negative symptoms and general subscales of the Positive and Negative Syndrome Scale (PANNS) (Shen et al., 2018). However, the mechanism by which statins reduce psychotic symptoms in these patients is still unclear, but it has been proposed that they can also protect patients with dementia from the onset of this type of manifestation (Shen et al., 2018).

There are reports of a possible association between pulmonary diseases and an increased risk of developing dementia (Liao et al., 2015; Peng et al., 2015). The use of short-acting bronchodilators, such as salbutamol and terbutaline, can cause symptoms such as anxiety, insomnia, motor restlessness and agitation, which may provide an explanation for the greater probability of patients who also have dementia being prescribed antipsychotics (Román, 2011).

The difference found for patients who received rivastigmine, who showed a lower probability of being prescribed an antipsychotic, may be because rivastigmine is used for patients with mild to moderate dementia (Cruz Jentoft and Hernández, 2014), whereas the use of memantine, whose indication of use is for more severe forms of the disease, may imply that patients are more symptomatic and therefore require antipsychotics (Fink et al., 2020).

In this study, it was found that having a concomitant diagnosis of dementia and anxiety markedly increases the likelihood of being prescribed an antipsychotic despite the risks associated with them, making the surveillance, symptom control and appropriate use of psychotropic drugs more important. In addition to the high frequency of comorbidities, studies conducted in people with dementia living in the community found that many behavioral symptoms are closely related to anxiety (Ferretti et al., 2001; Neville and Teri, 2011).

An expected finding was the relationship between psychotic disorders and being prescribed an antipsychotic, although all these drugs are considered potentially inappropriate prescriptions in patients with dementia, with the exception of cases in which there are no alternatives due to severe behavioral and psychotic symptoms (Magierski et al., 2020). The association between dementia and depression with a greater probability of being prescribed an antipsychotic has also been reported (Neil et al., 2003; Nemeroff, 2005; Byers and Yaffe, 2011). In this analysis, more than 45% of patients with dementia received some antidepressant, a finding that should incite clinicians to consider the need to add, or not, new drugs to this group of patients and the risks involved.

In this study, it was found that the concomitant use of anticonvulsants increased, by almost two-fold, the probability of being prescribed an antipsychotic, a result that may be due to the need to share the same objective to modulate symptoms such as anxiety, depression, apathy, and psychosis and control the behavioral symptoms of patients with dementia who do not respond to typical therapy; however, it cannot be ruled out that anticonvulsants are indicated for the control of seizures (Magierski et al., 2020).

In addition to anticonvulsants, the concomitant use of benzodiazepines seems to double the probability of being prescribed an antipsychotic; however, in this study, due to its cross-sectional observational nature, it cannot be established which was used first. Benzodiazepines are useful in the control of symptoms such as aggressiveness, sleep disturbances and anxiety, which may be associated with the fact that these patients have more behavioral disturbances (Donoghue and Lader, 2010; Dell'osso and Lader, 2013), but it cannot be ruled out that these drugs were used for indications such as panic attacks or anxiety disorders. However, the chronic use of benzodiazepines can worsen behavioral problems due to their amnesic and disinhibitory effects (Rayner et al., 2006).

This analysis has limitations characteristic of observational studies performed using a drug dispensing database. There was a lack of a review of medical records to verify the severity and time since onset of dementia and hence the need for certain drugs. The effectiveness of the therapy and the safety problems that were actually generated with the prescription of antipsychotics were also not evaluated. However, the results can be extrapolated to populations with similar insurance status and drug access characteristics.

Based on the above findings, it can be concluded that more than one-third of this group of Colombian patients diagnosed with dementia were being prescribed some antipsychotic that could potentially worsen their clinical picture and cause adverse events. The progressive increase in age, concomitant suffering from anxiety, depression or some psychotic disorder, and combined use with anticonvulsants and bronchodilators were associated with a greater probability of being prescribed an antipsychotic. Our findings can be used by clinicians and all those responsible for the care of patients with dementia to offer therapies with lower risks to these specific groups. Further studies are needed to explore the safety of these drugs in the context of real life.

## Data Availability

The database with all the information necessary to carry out the analyzes is deposited in protocols.io, https://doi.org/10.17504/protocols.io.bt7qnrmw.
